# A Novel System for the Quantification of the ADCC Activity of Therapeutic Antibodies

**DOI:** 10.1155/2017/3908289

**Published:** 2017-09-27

**Authors:** Christophe Lallemand, Feifei Liang, Flore Staub, Maud Simansour, Benoit Vallette, Lue Huang, Rosa Ferrando-Miguel, Michael G. Tovey

**Affiliations:** Biomonitor SAS, Villejuif Bio Park, 1 Mail du Professeur Georges Mathé, 94800 Villejuif, France

## Abstract

Novel ADCC effector cells expressing the V-variant or F-variant of Fc*γ*RIIIa (CD16a) and firefly luciferase under the control of a chimeric promoter incorporating recognition sequences for the principal transcription factors involved in Fc*γ*RIIIa signal transduction, together with novel target cells overexpressing a constant high level of the specific antigen recognized by rituximab, trastuzumab, cetuximab, infliximab, adalimumab, or etanercept, confer improved sensitivity, specificity, and dynamic range in an ADCC assay relative to effector cells expressing a NFAT-regulated reporter gene and wild-type target cells. The effector cells also contain a normalization gene rendering ADCC assays independent of cell number or serum matrix effects. The novel effector and target cells in a frozen thaw-and-use format exhibit low vial-to-vial and lot-to-lot variation in their performance characteristics reflected by CVs of 10% or less. Homologous control target cells in which the specific target gene has been invalidated by genome editing providing an ideal control and a means of correcting for nonspecific effects were observed with certain samples of human serum. The novel effector cells and target cells expressing noncleavable membrane-bound TNF*α* have been used to quantify ADCC activity in serum from patients with Crohn's disease treated with infliximab and to relate ADCC activity to drug levels.

## 1. Introduction

The activity of numerous therapeutic antibodies is mediated in part by immune-mediated effector cell function following binding of the variable regions of the antibody to a specific antigen on the surface of target cells and the interaction of the Fc moiety of the antibody with an Fc receptor on an immune effector cell [1]. This leads to clustering of the Fc receptors, tyrosine phosphorylation, calcium flux, and activation of several transcription factors in the immune effector cell(s). Antibody-mediated effector functions, in particular, antibody-dependent cellular cytotoxicity (ADCC), are considered to play a determining role in the action of a number of therapeutic antibodies including rituximab, trastuzumab, and cetuximab [2]. Traditional methods for quantifying ADCC activity are labor intensive and have a high level of inherent variability [3]. This is due to the use of primary human peripheral blood mononuclear cells (PBMC) or natural killer (NK) cells from different donors as the effector cells and the use of a complex endpoint which is difficult to standardize, namely, cytotoxicity. Although the traditional ^51^CR release assay has been largely replaced by alternative assays using 3-(4,5-dimethylthiazol-2-yl)-2,5 diphenyltetrazolium bromide (MTT), calcein-acetoxymethyl, or lactate dehydrogenase-release assays or various flow cytometric assays using Annexin V, propidium iodide, or 7-amino-actinomycin D, all these assays are subject to poor reproducibility, low sensitivity, and high levels of spontaneous release [4]. These limitations have been overcome in part by the use of engineered effector cells expressing the low-affinity Fc receptor, Fc*γ*RIIIA (CD16a), that responds to ligation of the Fc moiety of an antibody bound to the specific antigen expressed on target cells by activation of a NFAT responsive reporter gene construct [5, 6]; there is a need, however, for a method for the evaluation of ADCC activity with improved sensitivity and specificity. We describe herein a method for the quantification of ADCC activity based on the use of novel engineered effector cells carrying a reporter gene with binding sites for the principal transcription factors involved in Fc*γ*RIIIA signal transduction [7–11] that reflects more closely signaling from the Fc*γ*RIIIA receptor and exhibits improved sensitivity, specificity, and dynamic range when used to quantify ADCC activity relative to the reporter gene effector cell lines described previously [5, 6]. In addition, novel target cells have been developed that express a constant high level of the specific antigen recognized by a particular monoclonal antibody under the control of a strong constitutive promoter as well as the homologous control target cells in which the gene encoding the specific antigen has been inactivated by genome editing [12]. These engineered target cells and the homologous control cells allow differences in ADCC activity to be determined with precision and a high degree of specificity. The specific antigen negative target cells also provide a means for detecting and correcting of the nonspecific effects observed in the presence of certain samples of human serum.

## 2. Materials and Methods

### 2.1. Cells and Cell Culture

The human T-cell line Jurkat, Clone E6-1, the human B-cell line Raji, and the human embryonic kidney cell line HEK293 were purchased from the American Type Culture Collection (Manassas, VA). Jurkat and Raji cells were cultivated in RPMI 1640 medium supplemented with 10% heat-inactivated fetal bovine serum (FBS) (ThermoFisher Scientific, France). HEK293 cells were cultivated in Dulbecco's modified Eagle's minimal essential medium (DMEM) (ThermoFisher Scientific, France), supplemented with 10% heat-inactivated FBS (ThermoFisher Scientific, France). Thaw-and-use Fc*γ*RIIIA-V158 variant effector cells and Raji target cells were purchased from Promega (Madison, WI).

### 2.2. An Effector Cell Line Carrying an ADCC Responsive Reporter Gene

A series of constructs consisting of variants of the NFAT, NF*κ*B, AP1, CREB, and STAT recognition sequences was tested in transient transfection experiments, using the human T-cell line Jurkat, for their ability to drive transcription of the firefly luciferase (FL) reporter gene from a minimal SV40 promoter following induction with ionomycin (500 mM), TNF*α* (100 ng/ml), PMA (10 ng/ml), dibutyryl cAMP (100 *μ*M), and interferon alpha (100 IU/ml), either alone or in combination. These functional reporter gene constructs were then tested in an ADCC reaction in the presence of Jurkat and Raji cells at 1 : 1 ratio and rituximab (500 ng/ml). The results of these experiments were then used to design a synthetic chimeric promoter sequence (GGAAGCGAAAATGAAATTGACTGGGACTTTCCGGAGGAAAAACTGTTTCATACAGAAGGCGTGGATGTCCATATTAGGATGAGTCAGTGACGTCAGAGCCTGATTTCCCCGAAATGATGA) to regulate expression of the FL reporter gene construct. Jurkat cells were then transfected sequentially, using the Neon transfection system (ThermoFisher Scientific, France) according to the manufacturer's recommendations, with the FL reporter gene construct, an expression vector for Fc*γ*RIIIA, V-variant (InvivoGen, San Diego, CA) or the F-variant synthetized using standard techniques and inserted into the pUNO1 plasmid (InvivoGen, San Diego, CA), and the Nano-Luc® luciferase (NL) reporter gene under the control of a constitutive promoter. Stable clones were isolated and characterized for ADCC activity in the presence of a given therapeutic antibody and the appropriate target cells giving rise to the clonal iLite™ effector cell lines.

### 2.3. Establishment of CD20^+^ and CD20^−^ Target Cells

The gene encoding CD20 was invalidated in Raji cells using CRISPR/Cas9 genome editing [12]. Briefly, a guide RNA sequence (GGCCCTATTGCTATGCAATC) was cloned into the nuclease vector GeneArt CRISPR (ThermoFisher Scientific, France) to guide the Cas9 double-stranded DNA endonuclease to a specific site within exon 1 of the CD20 gene to obtain CD20^−^ Raji cells. CD20^−^ Raji cells were then transfected with a CD20 expression vector (InvivoGen, San Diego, CA) using the Neon transfection system (ThermoFisher Scientific, France) according to the manufacturer's recommendations. Positive clones were enriched using fluorescence-activated cell sorting and a FITC-labelled anti-CD20 monoclonal antibody (R&D Systems, Minneapolis, MN). Stable clones were isolated and characterized for ADCC activity in the presence of the iLite effector cells and rituximab giving rise to the CD20^+^ target cell line.

### 2.4. Establishment of ERBB2^+^ and ERBB2^−^ Target Cells

The gene encoding ERBB2 was invalidated in HEK293 cells using CRISPR/Cas9 genome editing. Briefly, a guide RNA sequence (TCATCGCTCACAACCAAGTG) was cloned into the nuclease vector GeneArt CRISPR (ThermoFisher Scientific, France) to guide the Cas9 double-stranded DNA endonuclease to a specific site within exon 6 of the *ERBB2* gene to isolate ERBB2^−^ HEK293 cells. HEK293^−^ cells were then transfected with an *ERBB2* expression vector (InvivoGen, San Diego, CA) using the FuGENE HD transfection reagent (Promega, Madison, WI). Positive clones were enriched using fluorescence-activated cell sorting and a FITC-labelled anti-ERBB2 monoclonal antibody (Abcam, Cambridge, UK). Stable clones were isolated and characterized for ADCC activity in the presence of the iLite effector cells and Herceptin™ (Roche, France) giving rise to the ERBB2^+^ HEK293 target cell line.

### 2.5. Establishment of EGFR^+^ and EGFR^−^ Target Cells

EGFR negative HEK293 cells [13] were transfected with the human EGFRa gene (InvivoGen, San Diego, CA) using the FuGENE HD transfection reagent (Promega, Madison, WI). Positive clones were enriched using fluorescence-activated cell sorting and a FITC-labelled anti-EGFR monoclonal antibody (R&D Systems, Minneapolis, MN). Stable clones were isolated and characterized for ADCC activity using the iLite effector cells and cetuximab (Erbitux™, Merck Serono, France) giving rise to the EGFR^+^ target cell line.

### 2.6. Establishment of mTNF*α*^+^ and mTNF*α*^−^ Target Cells

To establish a cell line that expresses membrane-bound noncleavable TNF*α*, site-directed mutagenesis was used to mutate the protease cleavage site to change amino acids AV to LL at position 76-77 of the gene encoding human TNF*α*. Briefly, nucleotides GCAGTC at positions 226 to 231 in the coding region of the native TNF*α* gene were replaced with nucleotides CTGTTC in the same position in a synthetic gene in which a Kozak sequence was also placed upstream of the start codon. To prevent noncleavable TNF*α* expressed on the cell surface binding to the TNF*α* receptor on neighboring cells, the TNFRSF1 gene encoding the TNF*α* receptor was invalidated in HEK293 cells using CRISPR/Cas9 genome editing. Briefly, two guide RNA sequences (ATATACCCCTCAGGGGTTAT and CACCGTGTGT GACTCCTGTG) were cloned into the nuclease vector GeneArt CRISPR (ThermoFisher Scientific, France) to guide the Cas9 double-stranded DNA endonuclease to a specific site within exon 2 of the TNFRSF1A gene located on chromosome 12 and a specific site within exon 3 of the TNFRSF1B gene located on chromosome 1, respectively, in order to isolate TNF*α*R^−^ HEK293 cells. Stable clones were isolated and characterized for ADCC activity in the presence of the iLite effector cells (V-variant) and infliximab (Remicade™, Janssen, France) giving rise to the mTNF*α* target cell line.

### 2.7. Stability of the Recombinant Effector and Target Cell Lines

A master cell bank (MCB) and a working cell bank (WCB) were prepared for the clonal effector cell lines and each of the clonal target cell lines. Each recombinant cell line was shown to be stable, as determined by both a constant response in an ADCC assay and stable growth characteristics, for at least 30 passages following their isolation.

### 2.8. Production of Assay-Ready Frozen Cells

Jurkat effector cells were frozen in RPMI 1640 medium and 20% FBS mixed 1 : 1 with cryoprotective medium (Lonza, France) at a concentration of 5.8 × 10^7^ cells/ml using standard techniques and stored at −80°C. Raji CD20^+^ and CD20^−^ target cells were frozen under the same conditions at a concentration of 1.9 × 10^7^ cells/ml. HEK293 ERBB2^+^ and ERBB2^−^ and EGFR^+^ and EGFR^−^ target cells were frozen under the same conditions at a concentration of 1.44 × 10^7^ cells/ml, and mTNF*α* and mTNF*α*^−^ target cells were frozen under the same conditions at a concentration of 9.6 × 10^6^ cells/ml.

### 2.9. Immunofluorescence and FACS Analysis

Cell surface expression of a specific antigen was visualized using an inverted fluorescent microscope (Evos, Life Technologies Inc.) and quantified using analytical flow cytometry (Accuri C6, BD, France). Fluorescence-activated cell sorting was carried out using a FacsAria (BD, France) and the appropriate antibody and isotype control. CD16 expression was determined using a biotin-labelled anti-CD16 antibody (Abcam, Cambridge, UK) and FITC-labelled streptavidin (AbCam, Cambridge, UK) and a mouse IgG1 k isotype control (BD Pharmingen, France). CD20 expression was determined using a FITC-labelled anti-CD20 monoclonal antibody (R&D Systems, Minneapolis, MN) and a PE-labelled mouse IgG1k isotype control antibody (BD Pharmingen, France). ERBB2 expression was determined using a phycoerythrin-labelled anti-ERBB2 monoclonal antibody (R&D Systems, Minneapolis, MN) and a FITC-labelled mouse IgG1 k isotype control (BD Pharmingen, France). EGFR expression was determined using a PE-labelled anti-EGFR/erbB1monoclonal antibody (R&D Systems, Minneapolis, MN) and a PE-labelled mouse IgG1 k isotype control (BD Pharmingen, France).

### 2.10. ADCC Reporter Gene Assay

For assays using cells in culture, Jurkat effector cells were harvested and resuspended in RPMI 1640 medium with 10% heat-inactivated FBS and 1.2 × 10^5^ cells in 20 *μ*l were added to each well of a white-sided microtiter plate (Perkin Elmer, Waltham, MA). Target cells at the appropriate E:T ratio in 20 *μ*l were then added to each well of the white-sided microtiter plate. Serial dilutions of the appropriate antibody were prepared in a separate microtiter plate, and 40 *μ*l was then added to the cells in a final volume of 80 *μ*l. The cells and antibody dilutions were incubated at 37°C in an atmosphere of 5% CO_2_ in air for the times indicated prior to the addition of the Nano-Glo® Dual-luciferase® reagent (Promega, Madison, WI) and the sequential quantification of FL and NL activity using a GloMax Explorer luminometer (Promega, Madison, WI). For assays using assay-ready frozen cells, Jurkat effector cells and the appropriate target cells were thawed rapidly in a 37°C water bath and diluted in RPMI 1640 medium with 10% heat-inactivated FBS to give the appropriate cell concentrations. Effector cells (1.2 × 10^5^) and target cells at the appropriate E:T ratio were added to each well of a white-sided microtiter plate, and serial dilutions of the appropriate antibody were prepared in a separate microtiter plate and then added to the cells in a final volume of 80 *μ*l. Subsequent steps were carried out in an identical manner to those for cells in culture. Results are expressed in relative luciferase units (RLU). Firefly RLU values can be normalized with respect to the Nano luciferase RLU values for each well to render results independent of cell concentration and nonspecific cytotoxicity. The raw data was imported into Prism GraphPad and analyzed using a four-parameter logistic (4PL) nonlinear regression curve.

### 2.11. Human Sera

Samples of serum from normal donors (Seralab, UK) or archival serum samples from patients with Crohn's disease treated with infliximab and monitored for the presence of antidrug neutralizing antibodies were randomly selected for evaluation in the present study. Informed consent was obtained for the use of serum samples for research purposes in accord with the Barts & Royal London Hospital Institutional Review Board guidelines. The nonspecific activation observed with some samples of human serum was corrected for by subtracting the value obtained in the presence of the iLite effector cells and the antigen negative target cells from that observed in the presence of the iLite effector cells and the antigen positive target cells using the following formula:

(1) $$\begin{array}{c} \frac{\left(E+T^{+}+drug+NHS\right)-\left(E+T^{-}+NHS\right)+\left(E+T^{+}\right)}{E+T^{+}}, \end{array}$$

or

(2) $$\begin{array}{c} \frac{\left(E+T^{+}+NHS\right)-\left(E+T^{-}+NHS\right)+\left(E+T^{+}\right)}{E+T^{+}}, \end{array}$$

where E is effector cells, T^+^ is antigen positive target cells, T^−^ is antigen negative target cells, and NHS is normal human serum sample.

## 3. Results

### 3.1. Establishment of a Reporter Gene Effector Cell Line

**Figure 1:**
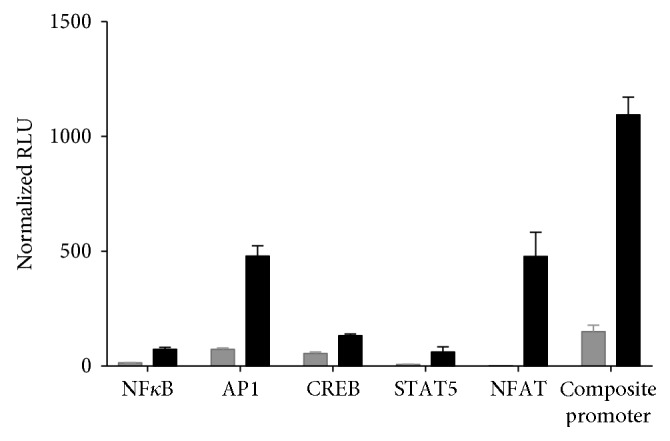
Activation of transcription factors implicated in CD16 signaling. Jurkat cells were cotransfected with a CD16a expression vector, the NL normalization gene, and a reporter gene construct for one of the transcription factors indicated in the figure and incubated in the presence of an equal concentration of wild-type Raji cells and 500 ng/ml of rituximab for 5 hours prior to the addition of Nano-Glo Dual-luciferase reagent (Promega, Madison, WI) and the sequential determination of FL and NL activity. Fold induction was calculated from the normalized FL RLU values observed in the presence of the Jurkat effector cells, Raji target cells, and rituximab after subtraction of the normalized FL RLU values obtained with Jurkat and Raji cells in the absence of rituximab.

To establish a reporter gene construct that responds optimally to ligation of the Fc*γ*RIIIA receptor (CD16a), a series of functional reporter gene constructs consisting of the recognition sequences for individual transcription factors implicated in CD16a signal transduction regulating expression of the firefly luciferase (FL) reporter gene was used to determine the response of a given transcription factor during an ADCC reaction. Briefly, the human T-cell line Jurkat was transiently cotransfected with a CD16a expression vector, the NL normalization gene, and a reporter gene construct for an individual transcription factor in the presence of rituximab and the human B-cell line Raji at a 1 : 1 concentration. Fold induction was determined at 5 hours after initiation of the ADCC reaction from the normalized FL RLU values observed in the presence of the Jurkat effector cells, Raji target cells, and rituximab after subtraction of the normalized RL RLU values obtained with Jurkat and Raji cells in the absence of rituximab (Figure 1). A significant level of activation of the NF*κ*B, AP-1, CREB, STAT4/5, and NFAT-regulated reporter gene constructs was observed 5 hours after initiation of an ADCC reaction (Figure 1).

Transient cotransfection of Jurkat cells with a CD16a expression vector and the FL reporter gene under the control of a chimeric promoter carrying binding sites for AP1, NF*κ*B, NFAT, CREB, and STAT 4/5 resulted in an additive increase in normalize FL activity determined at 5 hours postinduction (Figure 1). The results of these experiments were used to design a synthetic chimeric promoter sequence regulating expression of the FL reporter gene. Jurkat cells were then transfected sequentially, with the chimeric FL reporter gene construct, an expression vector for Fc*γ*RIIIA, V-variant or F-variant, and the NL reporter gene under the control of a constitutive promoter that allows drug-induced FL activity to be normalized with respect to the constitutive expression of NL. Stable clones were isolated and characterized for ADCC activity in the presence of rituximab and wild-type (WT) CD20 positive Raji target cells.

### 3.2. Establishment of Engineered Target Cells Expressing a High Constant Level of a Specific Antigen at the Cell Surface

#### 3.2.1. Establishment of CD20 Specific Target Cells

**Figure 2:**
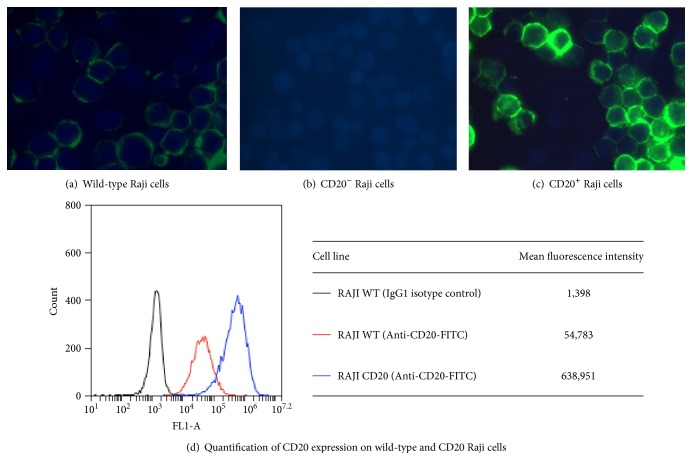
Expression of CD20 on the surface of Raji cells. (a, b, c) Cell surface expression of CD20 was visualized using an inverted fluorescent microscope and a FITC-labelled anti-CD20 monoclonal antibody. (d) Cell surface expression of CD20 was quantified using flow cytometry and a FITC-labelled anti-CD20 monoclonal antibody.

The gene encoding CD20 was invalidated in the human B-cell line Raji using CRISPR/Cas9 genome editing giving rise to CD20^−^ Raji cells. The CD20^−^ Raji cells were then transfected with the gene encoding CD20 under the control of a strong constitutive promoter; stable clones were isolated and characterized for ADCC activity in the presence of the iLite V-variant effector cells and rituximab giving rise to the CD20^+^ target cell line. WT Raji cells were found to express low variable levels of CD20 at the cell surface (Figure 2()) while CD20 expression was undetectable in the CD20^−^ target cells (Figure 2(b)). Overexpression of the CD20 gene resulted in a high constant level of CD20 at the surface of the CD20^+^ target cells (Figure 2(c)) that was approximately 16-fold higher than that of WT Raji cells (Figure 2(d)).

#### 3.2.2. Quantification of the ADCC Activity of Rituximab Using WT, CD20^+^, or CD20^−^ Raji Cells

**Figure 3:**
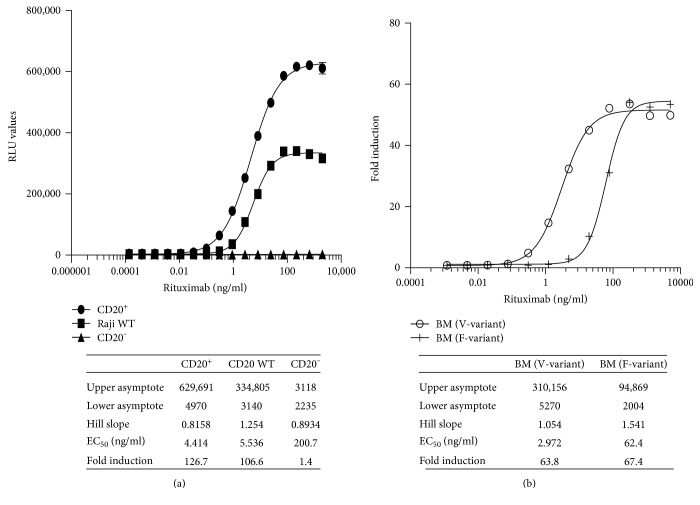
Comparison of the ADCC activity of rituximab using iLite effector cells V-variant or F-variant and wild type, CD20^+^, and CD20^−^ Raji target cells. iLite effector cells V-variant (1.2 × 10^5^ cells/well) were mixed with 4 × 10^4^ cells/well WT, CD20^+^, or CD20^−^ target cells (4 × 10^4^ cells/well) for 4 hours in the presence of increasing concentrations of rituximab. Results are expressed as FL activity in RLU (a). iLite effector cells, V-variant or F-variant (1.2 × 10^5^ cells/well) were mixed with CD20^+^ target cells (4 × 10^4^ cells/well) for 6 hours in the presence of increasing concentrations of rituximab (b) prior to the addition of Nano-Glo Dual-luciferase reagent (Promega, Madison, WI) and the sequential determination of FL and NL activity. Results are expressed as fold induction.

iLite CD16a V-variant effector cells (E) were incubated for 4 hours with WT, CD20^+^, or CD20^−^ Raji target cells (T) at an E:T ratio of 3 : 1 in the presence of increasing concentrations of rituximab prior to the sequential quantification of FL and NL activity. Results are expressed as relative luciferase units (RLU) and presented in the form of a 4-parametric logistic (4PL) plot (Figure 3(a) and associated table). The sensitivity (EC_50_), dynamic range, and RLU values of the ADCC assay carried out using iLite effector cells and CD20^+^ target cells were superior to that observed with the iLite effector cells and WT Raji cells (Figure 3(a)). No ADCC activity was detected when the iLite effector cells were incubated with CD20^−^ target cells and increasing concentrations of rituximab (Figure 3(a)).

A fixed concentration of iLite effector cells expressing either the V-variant or F-variant of CD16a was incubated for 6 hours with CD20^+^ Raji target cells at an E:T ratio of 3 : 1 in the presence of increasing concentrations of rituximab prior to the sequential quantification of FL and NL activity. As expected, the sensitivity of detection of the ADCC activity of rituximab was significantly greater using iLite effector cells expressing the V-variant of CD16a than that observed using iLite effector cells expressing the F-variant of CD16a even though the dynamic range and RLU values obtained were similar (Figure 3(b)).

#### 3.2.3. Establishment of the Optimal Effector Target Cell Ratio

**Figure 4:**
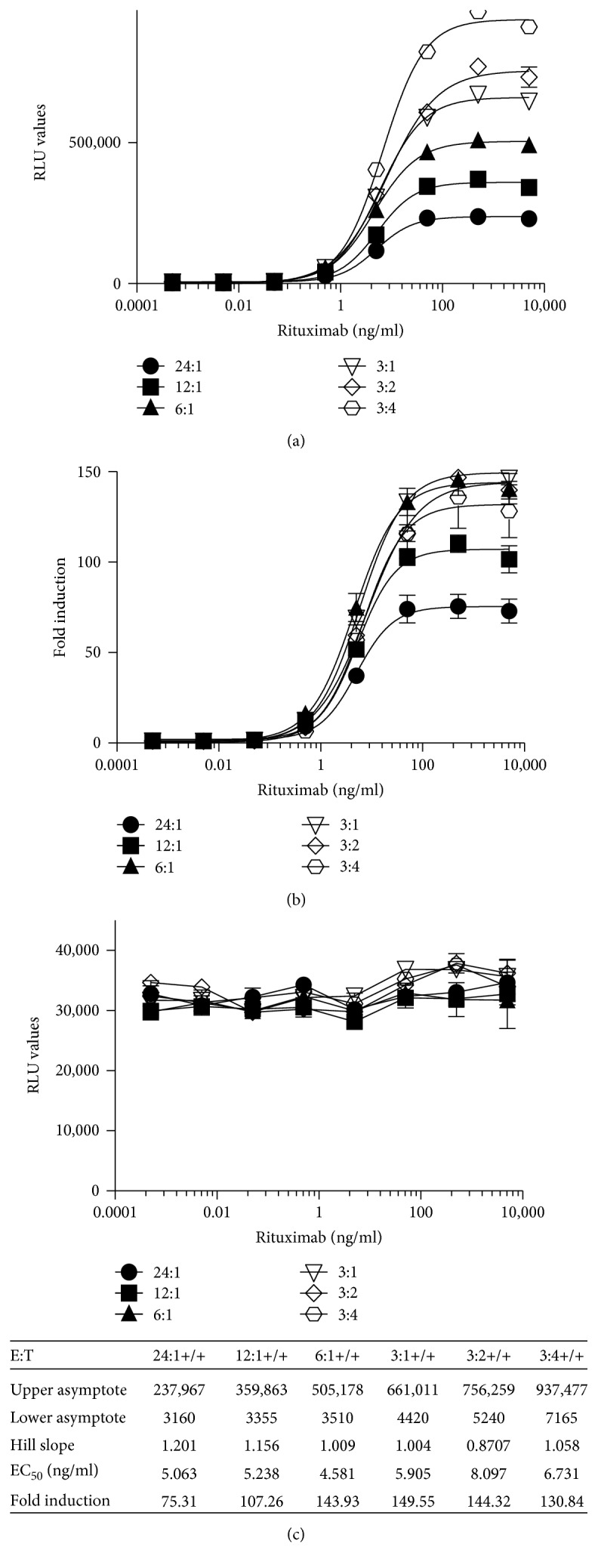
Quantification of the ADCC activity of rituximab using frozen ready-to-use iLite effector cells and CD20^+^ target cells. Frozen RTU iLite effector cells (1.2 × 10^5^ cells/well) and CD20^+^ target cells were thawed rapidly and mixed at the E:T ratio indicated and incubated for 4 hours at 37°C in the presence of increasing concentrations of rituximab prior to the addition of Nano-Glo Dual-luciferase reagent (Promega, Madison, WI) and the sequential determination of FL and NL activity. Results are expressed as FL activity in RLU (a) or as fold induction (b) and as NL activity in RLU (c).

To determine the optimal effector target cell ratio (E:T) for the quantification of the ADCC activity of rituximab, a fixed concentration of the iLite effector cells (E) that yielded a readily detectable level of FL expression was incubated with varying concentrations of CD20^+^ target cells (T). The optimal E:T ratio for iLite effector cells that gave readily measurable levels of FL activity while at the same time yielding an optimal dynamic range after 4 hours incubation of the effector cells and target cells in the presence of increasing concentrations of rituximab was found to be 3 : 1 using either effector cells and CD20^+^ target cells in culture (data not shown) or ready-to-use (RTU) effector cells and CD20^+^ target cells frozen separately, thawed, and used immediately without preincubation (Figures 4(a) and 4(b)). The EC_50_ for the ADCC activity of rituximab at an E:T ratio of 3 : 1 was 5.9 ng/ml, and the dynamic range of the assay was 150-fold for RTU cells (Figure 4(b)).

**Figure 5:**
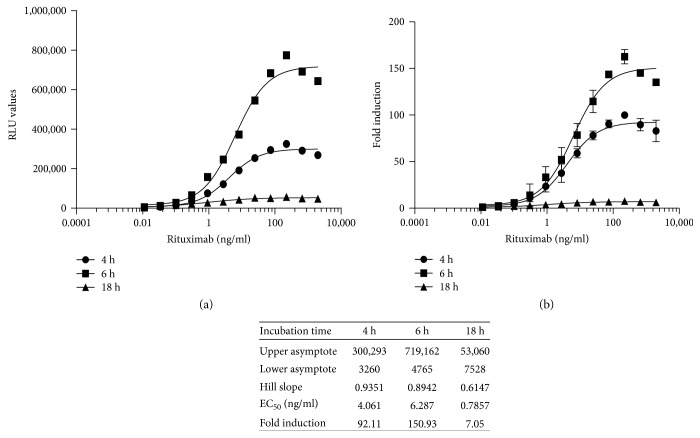
Quantification of the ADCC activity of rituximab using RTU iLite effector cells and CD20^+^ target cells: Effect of incubation time. RTU iLite effector cells (1.2 × 10^5^ cells/well) and CD20^+^ target cells were mixed at an E:T ratio of 3 : 1 and incubated for 4, 6, or 18 hours at 37°C in the presence of increasing concentrations of rituximab prior to the addition of Nano-Glo Dual-luciferase reagent (Promega, Madison, WI) and the sequential determination of FL and NL activity. Results are expressed as FL activity in RLU (a) or fold induction (b).

NL expression did not vary as a function of either rituximab concentration or the E:T ratio for either cells in culture or RTU cells (Figure 4(c)). Although incubation of the effector cells and CD20^+^ target cells for 6 hours increased both the FL RLU values and the fold induction, the sensitivity was lower at 6 hours for both cells in culture and RTU cells (Figure 5(a and b)). Incubation of the effector cells and CD20^+^ target cells for 18 hours adversely affected both the RLU values obtained (Figure 5, a) and fold induction (Figure 5, b).

**Figure 6:**
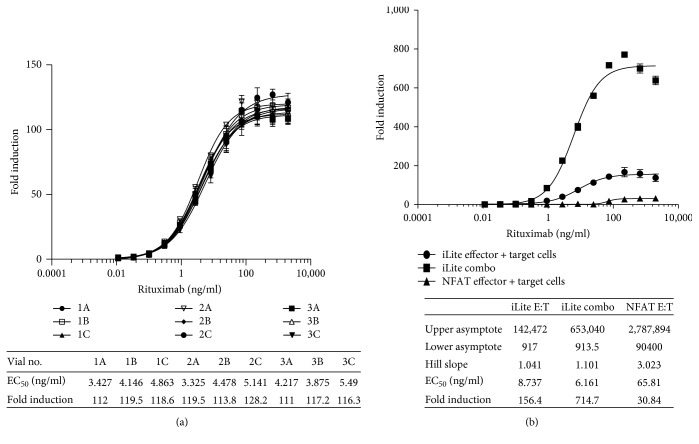
Quantification of the ADCC activity of rituximab. Individual vials of frozen iLite effector cells (1.2 × 10^5^ cells/well) and vials of frozen CD20^+^ target cells from 3 different production lots (1 to 3) were thawed, mixed at E:T ratio of 3 : 1, and incubated for 4 hours at 37°C in the presence of increasing concentrations of rituximab in a 384-well microtiter plate prior to the addition of Nano-Glo Dual-luciferase reagent (Promega, Madison, WI) and the sequential determination of FL and NL activity (a). RTU frozen iLite effector cells and CD20^+^ target cells frozen separately or together at E:T ratio of 3 : 1 and incubated for 4 hours or thaw-and-use NFAT effector cells and CD20^+^target cells at an E:T ratio of 6 : 1 and incubated for 6 hours at 37°C in the presence of increasing concentrations of rituximab (b). Results are expressed as fold induction relative to the control sample consisting of effector cells and target cells without rituximab.

A low degree of vial-to-vial and lot-to-lot variation was observed using frozen RTU effector and target cells resulting in precise and reproducible ADCC assays as illustrated in Figure 6(a) showing the results for individual vials of frozen RTU cells from 3 different production lots assayed on a 384-well microtiter plate. The associated table in Figure 6(a) shows the principal parameters of a 4PL plot.

Preincubation of iLite effector cells and CD20^+^ target cells at the optimal E:T ratio of 3 : 1 for 2 hours at 37°C in the absence of rituximab prior to freezing the effector cells and target cells together in the same vial, once thawed and used to quantify the ADCC activity of rituximab, was found to increase both the sensitivity and dynamic range of the assay (Figure 6(b)).

**Figure 7:**
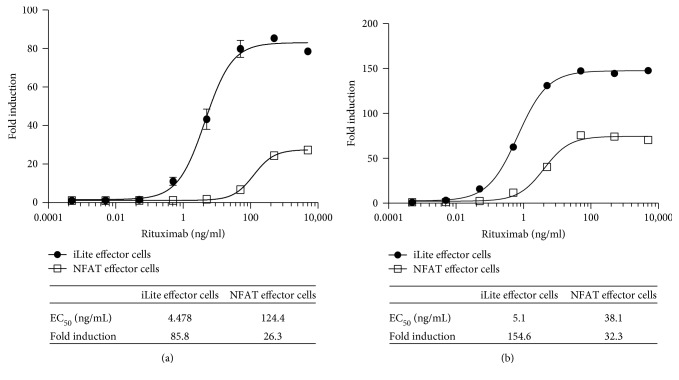
Quantification of the ADCC activity of rituximab using iLite effector cells and CD20^+^ target cells or NFAT effector cells and WT Raji cells. Assay-ready frozen iLite effector cells (1.2 × 10^5^ cells/well) or thaw-and-use NFAT effector cells (1.2 × 10^5^ cells/well) and frozen assay-ready CD20^+^ target cells were mixed at an E:T ratio of 1 : 3 and 1 : 6, respectively, and incubated for 4 (a) or 6 hours (b) at 37°C in the presence of increasing concentrations of rituximab prior to the addition of Nano-Glo Dual-luciferase reagent (Promega, Madison, WI) or Bio GLO, respectively, and the sequential determination of FL and NL activity or FL activity alone. Results are expressed as fold induction relative to the control sample without rituximab.

Both the dynamic range and EC_50_ of the ADCC assay using frozen ready-to-use iLite effector cells and CD20^+^ target cells were superior to those obtained using thaw-and-use NFAT responsive effector cells when used in conjunction with either WT (Figure 7(a)) or CD20^+^ (Figures 6(b) and 7(b)) Raji target cells. Thus, a dynamic range of 86-fold and 148-fold was observed after 4-hour incubation of the iLite effector cells with WT (Figure 7(a)) or CD20^+^ Raji target cells (Figure 7(b)) at an E:T ratio of 3 : 1 in the presence of increasing concentrations of rituximab compared with a dynamic range of 26-fold and 32-fold observed after 6-hour incubation of the NFAT effector cells with WT (Figure 7(a)) or CD20^+^ target cells (Figure 7(b)) at an E:T ratio of 6 : 1 as recommended by the manufacturer. An EC_50_ of 4.5 and 5.0 ng/ml of rituximab was observed at 4 and 6 hours, respectively, using iLite effector cells and CD20^+^ target cells. In contrast, an EC_50_ of 124 and 38 ng/ml of rituximab was observed at 4 and 6 hours, respectively, using the NFAT effector cells and CD20^+^ target cells using an E:T ratio of 6 : 1 recommended by the manufacturer (Figures 7(a) and 7(b) and associated tables).

#### 3.2.4. Quantification of the ADCC Activity of Rituximab in the Presence of Normal Human Serum

**Figure 8:**
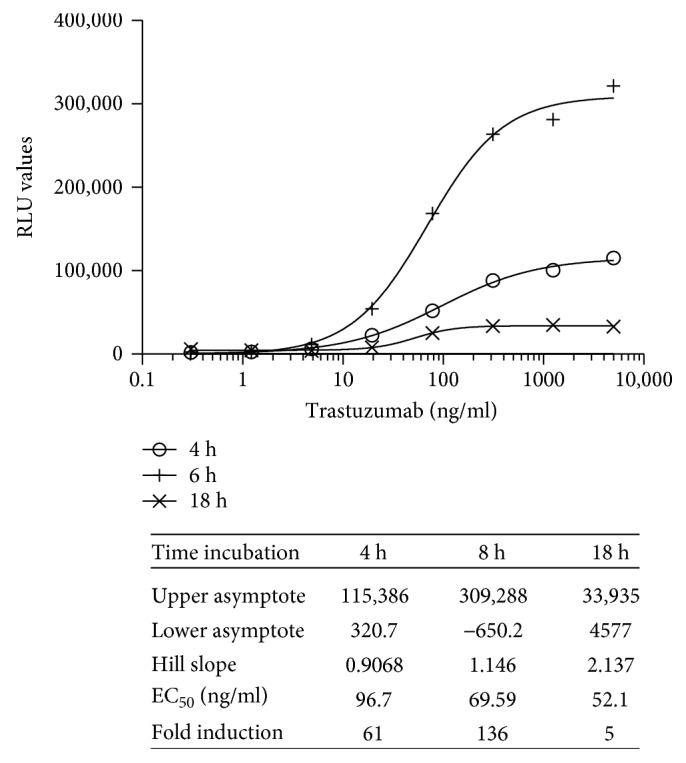
Quantification of the ADCC activity of trastuzumab using iLite effector cells (V-variant) and ERBB2^+^ HEK293 targets: effect of incubation time. RTU iLite effector cells (1.2 × 10^5^ cells/well) and ERBB2^+^ HEK293 target cells were mixed at an E:T ratio of 4 : 1 and incubated for 4, 6, or 18 hours at 37°C in the presence of increasing concentrations of trastuzumab prior to the addition of Nano-Glo Dual-luciferase reagent (Promega, Madison, WI) and the sequential determination of FL and NL activity. Results are expressed as FL activity in RLU.

**Figure 9:**
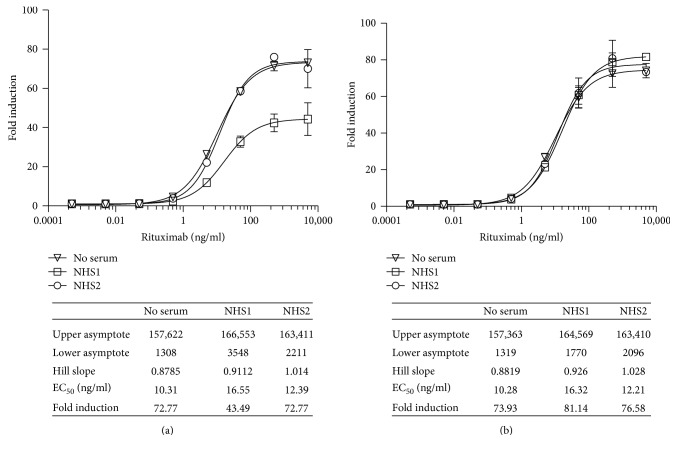
Quantification of the ADCC activity of rituximab using iLite effector cells and CD20^+^ Raji target cells in the presence of normal human serum. Frozen RTU iLite effector cells (1.2 × 10^5^ cells/well), CD20^−^, and CD20^+^ target cells were thawed rapidly, and the effector cells were mixed with the CD20^−^ or CD20^+^ target cells at an E:T ratio of 3 : 1 and incubated for 4 hours at 37°C in the presence of RPMI 1640 medium +10% FBS either alone or together with a 1/20 final dilution of samples of human serum from normal donors in the presence of increasing concentrations of rituximab prior to the addition of Nano-Glo Dual-luciferase reagent (Promega, Madison, WI) and the sequential determination of FL and NL activity. Results are expressed as fold induction relative to the control sample without rituximab. (a) Fold induction and (b) fold induction following subtraction of the values obtained with the iLite effector cells and the CD20^−^ target cells as described in Materials and Methods.

Vials of RTU iLite effector cells and vials of RTU CD20^+^ target cells were thawed, and the effector cells and target cells were mixed at an E:T ratio of 3 : 1 and incubated for 4 hours in the presence of increasing concentrations of rituximab in either culture medium +10% FBS alone or together with a 1/20 final dilution of a pool of normal human serum (NHS) or sera from individual normal donors prior to the sequential determination of FL and NL activity. In most cases, the presence of either a pool of normal human serum or sera from individual normal donors had no significant effect on the response of the iLite effector cells and CD20^+^ target cells (Figure 8). Approximately 1 in 10 of the sera from normal individual donors tested did, however, exert a nonspecific activation of the iLite effector cells in the presence of both the CD20^+^ target cells and the CD20^−^ negative target cells in the absence of rituximab indicating the nonspecific nature of the activation. The nonspecific effect observed with certain samples of normal human serum could be corrected, as described in Materials and Methods, by subtracting the value obtained in the presence the iLite effector cells and the CD20^−^ negative Raji target cells from that observed in the presence of the iLite effector cells and the CD20^+^ positive Raji target cells (Figures 9(a) and 9(b)).

#### 3.2.5. Establishment of Target Cells Expressing a High Constant Level of ERBB2

The gene encoding ERBB2 was invalidated in HEK293 cells using genome editing to isolate ERBB2 negative HEK293 (ERBB2^−^ HEK293) target cells. ERBB2^−^ HEK293 cells were then transfected with an *ERBB2* expression vector, and stable clones were isolated and characterized for ADCC activity in the presence of the iLite effector cells and trastuzumab (Herceptin) giving rise to the ERBB2^+^ HEK293 target cell line. A low variable level of ERBB2 expression was detected on the surface of wild-type HEK293 cells while ERBB2 expression was undetectable in ERBB2^−^ HEK293 cells in contrast to the constant high level of expression detected on the surface of ERBB2^+^ HEK293 cells following labelling with a FITC-labelled anti-ERBB2 monoclonal antibody (data not shown).

#### 3.2.6. Quantification of the ADCC Activity of Trastuzumab Using WT, ERBB2^−^, or ERBB2^+^ HEK293 Cells

**Figure 10:**
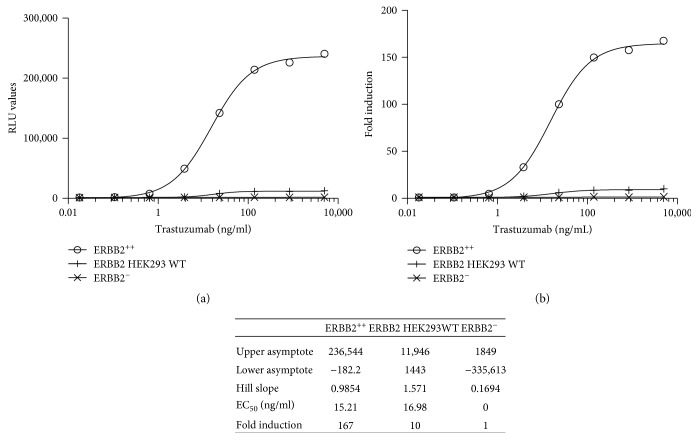
Comparison of the ADCC activity of WT, ERBB2^+^, and ERBB2^−^ HEK293 cells. iLite effector cells (1.2 × 10^5^ cells/well) were mixed with 3 × 10^4^ WT, ERBB2^+^, or ERBB^−^ target cells for 6 hours in the presence of increasing concentrations of trastuzumab prior to the addition of Nano-Glo Dual-luciferase reagent (Promega, Madison, WI) and the sequential determination of FL and NL activity. Results are expressed as RLU (a) or fold induction (b).

A fixed concentration of the iLite effector cells that gave a readily detectable level of FL expression was incubated for 6 hours with WT or ERBB2^+^ target cells at an E:T ratio of 1 : 1 in the presence of increasing concentrations of trastuzumab (Figure 10). Both the EC_50_ and dynamic range of the ADCC assay observed with the iLite effector cells and ERBB2^+^ HEK293 target cells were superior to those observed with the iLite effector cells and WT HEK293 cells (Figure 10). No ADCC activity was detected when the iLite effector cells were incubated with ERBB2^−^ HEK293 target cells and increasing concentrations of trastuzumab (Figure 10(a and b)).

#### 3.2.7. Establishment of the Optimal Effector Target Cell Ratio

To determine the optimal effector target cell ratio (E:T) for the quantification of the ADCC activity of trastuzumab, a fixed concentration of the V-variant of iLite effector cells that yielded a readily detectable level of FL expression was incubated with varying concentrations of ERBB2^+^ HEK293 target cells. The optimal E:T ratio that gave readily measurable levels of FL activity while at the same time yielding an optimal dynamic range after 6-hour incubation of the iLite effector cells and ERBB2^+^ HEK293 target cells in the presence of increasing concentrations of trastuzumab was found to be 4 : 1 both for cells in culture and for RTU effector cells and RTU target cells thawed and used immediately without preincubation (data not shown). Incubation of the effector cells and ERBB2^+^ target cells for 18 hours resulted in a decrease in both RLU values and the dynamic range of the assay relative to that observed at 6 hours (Figure 8). In contrast, the EC_50_ of the assay was decreased at 18 hours (Figure 8 and associated table).

**Figure 11:**
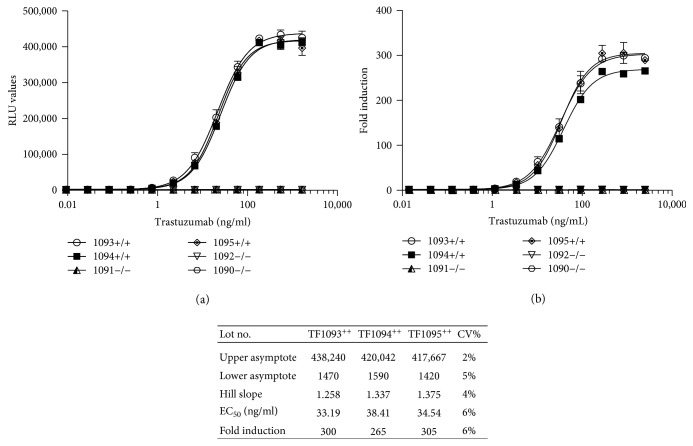
Quantification of the ADCC activity of trastuzumab using RTU iLite effector cells and ERBB2^+^ HEK293 target cells. Individual vials of frozen RTU iLite effector cells and vials of frozen RTU ERBB2^+^ or ERBB2^−^ HEK293 target cells from 3 different production lots were thawed, mixed at E:T ratio of 4 : 1, and incubated for 6 hours at 37°C in the presence of increasing concentrations of trastuzumab prior to the addition of Nano-Glo Dual-luciferase reagent (Promega, Madison, WI) and the sequential determination of FL and NL activity. Results are expressed as RLU (a) or fold induction relative to the control sample consisting of effector and target cells without trastuzumab (b).

Frozen RTU iLite effector cells and RTU ERBB2^+^ or ERBB2^−^ HEK293 target cells when thawed and used immediately at an E:T ratio of 4 : 1 without preincubation were found to yield results comparable to those obtained using cells in culture (data not shown). A low degree of vial-to-vial and lot-to-lot variation was observed using frozen RTU iLite effector cells and RTU ERBB2^+^ HEK293 target cells resulting in precise and reproducible ADCC assays as illustrated in Figure 11(a and b) and the associated table showing the principal parameters of a 4PL plot for individual vials of frozen RTU cells from 3 different production lots. No detectable ADCC activity was detected using RTU iLite effector cells and RTU ERBB2^−^ HEK293 target cells.

**Figure 12:**
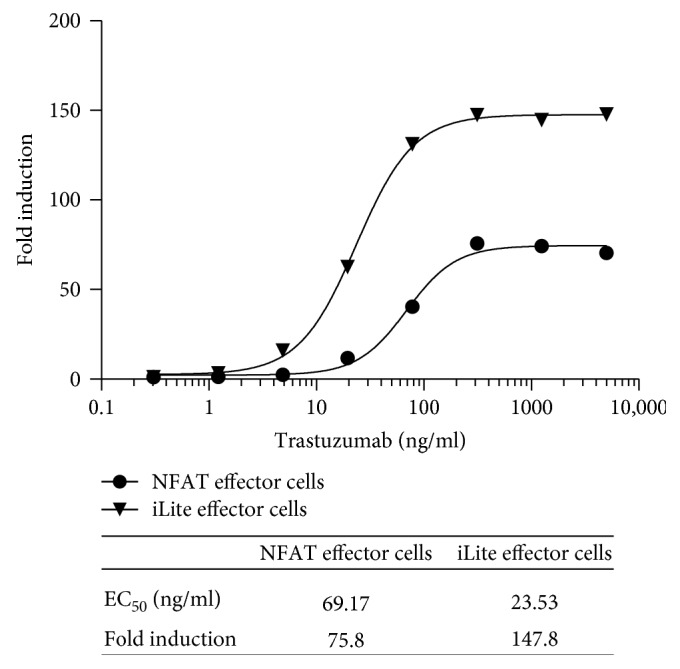
Quantification of the ADCC activity of trastuzumab using frozen RTU iLite effector cells and ERBB2^+^ HEK293 target cells or thaw-and-use NFAT effector cells and SK-BR-3 target cells. RTU frozen iLite effector cells V-variant (1.2 × 10^5^ cells/well) or thaw-and-use NFAT effector cells (1.2 × 10^5^ cells/well) and frozen RTU ERBB2^+^ HEK293 target cells or thaw-and-use NFAT effector cells (0.75 × 10^5^ cells/well) and SK-BR-3 target cells at an E:T ratio of 4 : 1 and 6 : 1, respectively, were incubated for 6 hours at 37°C in the presence of increasing concentrations of trastuzumab prior to the addition of Nano-Glo Dual-luciferase reagent (Promega, Madison, WI) or Bio Glo, respectively, and the sequential determination of FL and NL activity or FL activity alone. Results are expressed as fold induction relative to the control sample without trastuzumab.

NL expression did not vary as a function of either trastuzumab concentration or the E:T ratio for either cells in culture or RTU cells (data not shown). Both the dynamic range and EC_50_ of the assay for the quantification of the ADCC activity assay of trastuzumab using the iLite effector cells and ERBB2^+^ HEK293 target cells were superior to those obtained using a NFAT responsive reporter gene effector cell line and SK-BR-3 target cells (Figure 12).

#### 3.2.8. Quantification of the ADCC Activity of Trastuzumab in the Presence of Normal Human Serum

**Figure 13:**
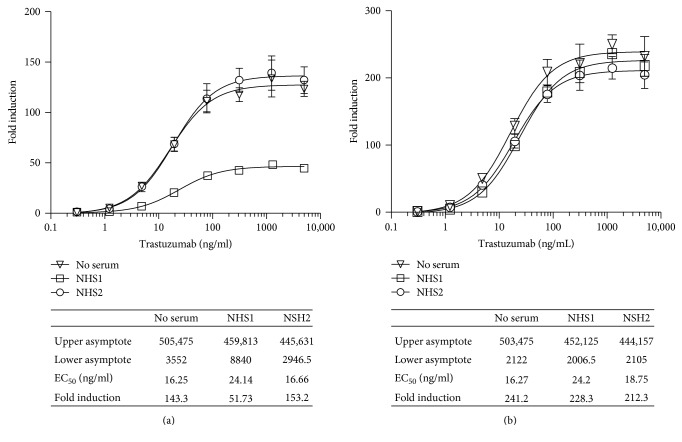
Quantification of the ADCC activity of Herceptin using iLite effector cells and ERBB2^+^ target cells in the presence of normal human serum. Frozen RTU iLite effector cells V-variant (1.2 × 10^5^ cells/well), ERBB2^−^, and ERBB2^+^ target cells were thawed rapidly, and the effector cells were mixed with the ERBB2^−^ or ERBB2^+^ target cells at an E:T ratio of 4 : 1 and incubated for 6 hours at 37°C in the presence of RPMI 1640 medium +10% FBS alone or together with a 1/20 final dilution of samples of human serum from normal donors in the presence of increasing concentrations of Herceptin prior to the addition of Nano-Glo Dual-luciferase reagent b (Promega, Madison, WI) and the sequential determination of FL and NL activity. Results are expressed as fold induction relative to the control sample without trastuzumab. (a) Fold induction and (b) fold induction following subtraction of the values obtained with the iLite effector cells and the ERBB2^−^ target cells.

Vials of RTU iLite effector cells and vials of RTU ERBB2^+^ HEK293 target cells were thawed, and the effector cells and target cells were mixed at an E:T ratio of 4 : 1 and incubated for 6 hours in the presence of increasing concentrations of trastuzumab in either culture medium +10% FBS alone or together with a 1/20 final dilution of sera from individual normal donors prior to the sequential determination of FL and NL activity. Although in most cases the presence of normal human serum from normal individual donors had no significant effect on the response of the iLite effector cells and ERBB2^+^ target cells in the absence of trastuzumab, approximately 10% of the sera tested from normal donors did exhibit a nonspecific activation that could be corrected by subtracting value obtained in the presence the iLite effector cells and the ERBB2 negative HEK293 target cells from that observed in the presence of the iLite effector cells and the ERBB2 positive HEK293 target cells as described in the Materials and Methods (Figures 13(a) and 13(b)).

#### 3.2.9. Establishment of Target Cells Expressing a High Constant Level of EGFR

EGFR negative HEK293 cells [14] were transfected with a human EGFRa expression vector stable clones that were isolated and characterized for ADCC activity using the iLite effector cells and cetuximab giving rise to the EGFR^+^ target cell line. EGFR expression was undetectable in WT HEK293 cells in contrast to the constant high levels detected in HEK293 EGFR^+^ cells following labelling with a FITC-labelled anti-EGFR monoclonal antibody (data not shown).

**Figure 14:**
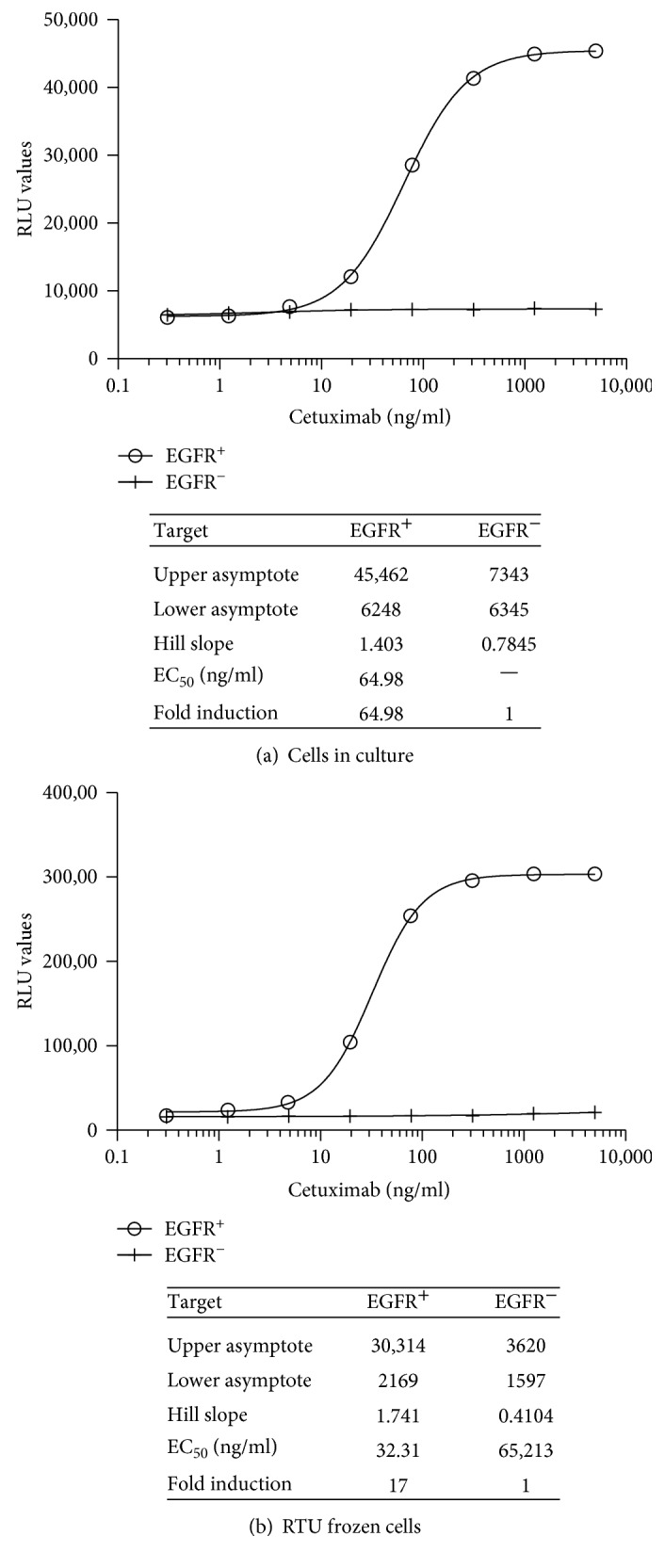
Quantification of the ADCC activity of cetuximab using iLite Effector cells and EGFR^+^ or EGFR^−^ target cells. iLite effector cells V-variant (1.2 × 10^5^ cells/well) and/or EGFR^+^ or EGFR^−^ EK293 target in culture or RTU iLite effector cells and EGFR^+^ or EGFR^−^ HEK293 target cells were mixed at an E:T ratio of 4 : 1 and incubated for 6 hours at 37°C in the presence of increasing concentrations of cetuximab prior to the addition of Nano-Glo Dual-luciferase reagent (Promega, Madison, WI) and the sequential determination of FL and NL activity. (a) Cells in culture and (b) RTU cells.

An E:T ratio of 4 : 1 iLite effector cells per EGFR^+^ target cell was found to give readily detectable level of FL expression and an optimal dynamic range after 6-hour incubation of the effector cells and target cells in the presence of increasing concentrations of cetuximab (Erbitux) for both cells in culture or frozen RTU cells (data not shown). Frozen RTU iLite effector cells and RTU EGFR^+^ target cells thawed and used immediately at an E:T ratio of 4 : 1 without preincubation were found to exhibit an increased sensitivity (EC_50_ 32 ng/ml) relative to that obtained using cells in culture (EC_50_ 65 ng/ml) in contrast to lower RLU values and a lower dynamic range (Figure 14). NL expression did not vary as a function of either cetuximab concentration or the E:T ratio for either cells in culture or RTU cells (data not shown). No ADCC activity was detected when the iLite effector cells in culture or RTU iLite effector cells were incubated with EGFR^−^ HEK293 target cells and increasing concentrations of cetuximab (Figures 14(a) and 14(b)).

**Figure 15:**
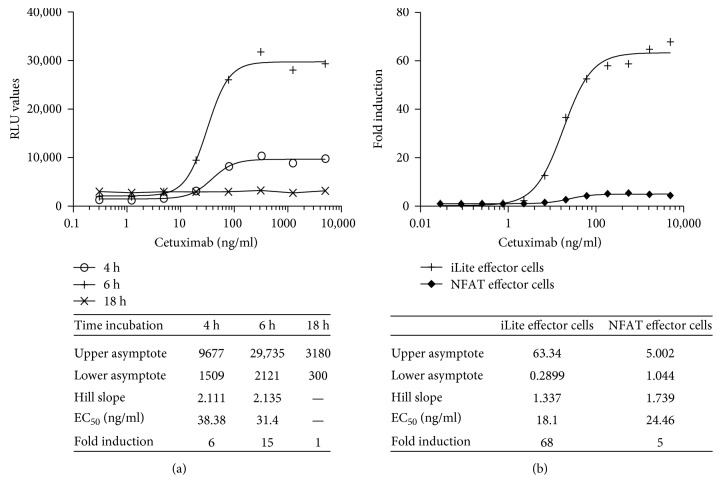
Quantification of the ADCC activity of cetuximab using iLite effector cells (V-variant) and EGFR^+^ HEK293 target cells: effect of incubation time. (a) RTU iLite effector cells V-variant (1.2 × 10^5^ cells/well) and RTU EGFR^+^ HEK293 target cells were mixed at an E:T ratio of 4 : 1 and incubated for 4, 6, or 18 hours at 37°C in the presence of increasing concentrations of cetuximab prior to the addition of Nano-Glo Dual-luciferase reagent (Promega, Madison, WI) and the sequential determination of FL and NL activity. Results are expressed as FL activity in RLU. (b) iLite effector cells V-variant (1.2 × 10^5^ cells/well) or NFAT responsive effector cells V-variant cells and EGFR^+^ HEK293 target cells were mixed at an E:T ratio of 4 : 1 and incubated for 6 at 37°C in the presence of increasing concentrations of cetuximab prior to the addition of Nano-Glo Dual-luciferase reagent (Promega, Madison, WI) and the sequential determination of FL and NL activity. Results are expressed as fold induction relative to the control without cetuximab.

Incubation of iLite effector cells and EGFR^+^ target cells for 6 hours was found to give a maximum dynamic range while incubation for 18 hours markedly reduced both RLU values and fold induction both for cells in culture and RTU target cells thawed and used immediately (Figures 15(a) and 15(b)).

A low degree of vial-to-vial, lot-to-lot, and plate-to-plate variation was observed using frozen RTU iLite effector and RTU EGFR^++^ target cells resulting in precise and reproducible ADCC assays (data not shown).

Both the EC_50_ and dynamic range of the ADCC assay using the iLite effector cell line were superior to those obtained using a NFAT responsive reporter gene effector cell line when used in conjunction with EGFR^+^ target cells (Figure 15(b)).

#### 3.2.10. Establishment of Target Cells Expressing a High Constant Level of mTNF*α*

To establish a cell line that expresses membrane-bound noncleavable TNF*α* suitable for use as a target cell for the quantification of the ADCC activity of TNF*α* antagonists, site-directed mutagenesis was used to mutate the protease cleavage in the gene encoding human TNF*α* to change amino acids AV to LL at positions 76-77. To prevent noncleavable TNF*α* expressed on the cell surface binding to the TNF*α* receptor present on the surface of neighboring cells resulting in cell death, the TNF*α*RI receptor was invalidated in HEK293 cells expressing noncleavable TNF*α* using genome editing; stable clones were isolated and characterized for ADCC activity in the presence of the iLite effector cells and infliximab (Remicade) giving rise to the mTNF*α* target cell line. A constant high level of mTNF*α* was detected on the surface of the mTNF*α*^+^ target cells following treatment with infliximab and a labelling with a FITC-labelled secondary anti-human IgG1 antibody while no labelling was observed with the isotype control primary antibody. In contrast, TNF*α* was undetectable on the surface of WT HEK293 cells following treatment with infliximab and a FITC-labelled secondary anti-human IgG1 antibody or with the isotype control antibody (data not shown).

**Figure 16:**
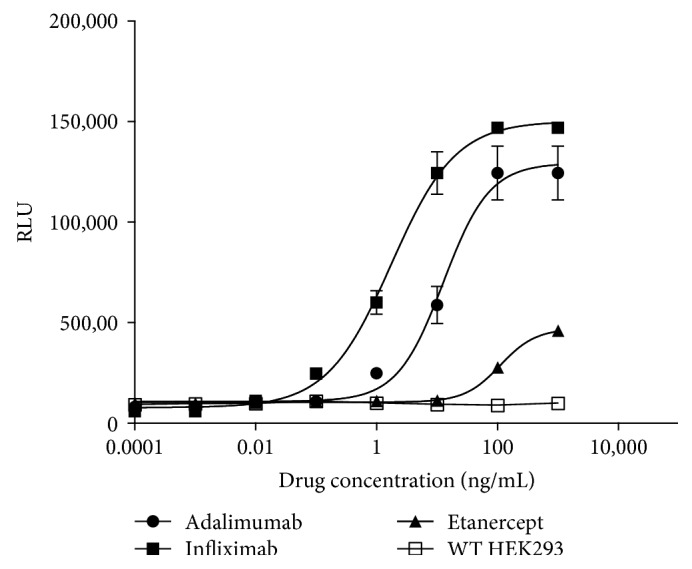
Quantification of the ADCC activity of TNF antagonists using iLite effector cells and mTNF*α*^+^ or mTNF*α*^−^ WT HEK293 target cell. iLite effector cells (1.2 × 10^5^ cells/well) were incubated with 2 × 10^4^ mTNF*α* target cells for 6 hours in the presence of increasing concentrations of infliximab, adalimumab, or etanercept prior to the addition of Nano-Glo Dual-luciferase reagent (Promega, Madison, WI) and the sequential determination of FL and NL activity. Results are expressed as FL activity in RLU.

A fixed concentration of the iLite V-variant effector cells in culture that gave a readily detectable level of FL expression was incubated for 6 hours with mTNF*α*^+^ or WT HEK293 target cells at an E:T ratio of 6 : 1 in the presence of infliximab. A dose-dependent increase in ADCC activity was observed with increasing concentrations of infliximab (Figure 16). In contrast, no ADCC activity was detected when the iLite effector cells were incubated with WT HEK293 cells at an E:T ratio of 6 : 1 in the presence of increasing concentrations of infliximab (Figure 16). A dose-dependent increase in ADCC activity was also observed when the iLite effector cells were incubated for 6 hours with mTNF*α*^+^ target cells and increasing concentrations of adalimumab (Humira™) and etanercept (Enbrel™) (Figure 16). An E:T ratio of 6 : 1 was found to give readily measurable levels of FL activity while at the same time yielding an optimal dynamic range after 6 hours incubation of the iLite effector cells and mTNF*α* HEK293 target cells in the presence of increasing concentrations of infliximab for both cells in culture and RTU cells (data not shown).

**Figure 17:**
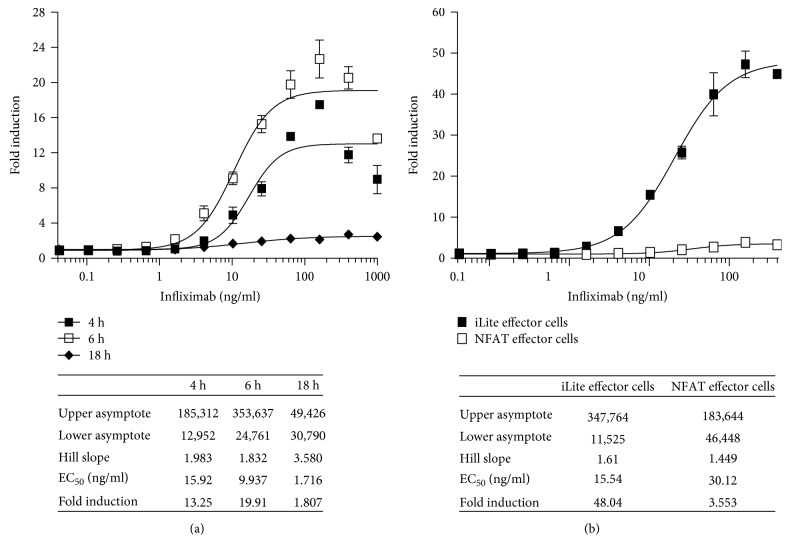
Quantification of the ADCC activity of infliximab using ready-to-use iLite effector cells or thaw-and-use NFAT effector cells and mTNF*α* target cells. RTU iLite effector cells (1.2 × 10^5^ cells/well) and mTNF*α* target cells were mixed at an E:T ratio of 6 : 1 and incubated for 4, 6, or 18 hours at 37°C in the presence of increasing concentrations of infliximab (a), RTU iLite effector cells (1.2 × 10^5^ cells/well) or thaw-and-use NFAT responsive effector cells (0.75 × 10^5^ cells/well), and RTU mTNF*α* target cells were mixed at an E:T ratio of 6 : 1 and incubated for 6 hours at 37°C in the presence of increasing concentrations of infliximab (b) prior to the addition of Nano-Glo Dual-luciferase reagent (Promega, Madison, WI) and the sequential determination of FL and NL activity. Results are expressed as fold induction of FL activity.

Incubation of RTU iLite effector cells and mTNF*α* target cells for 18 hours markedly decreased both the RLU values obtained and the dynamic range of the assay, although the sensitivity of the assay increased relative to that observed at 6 hours (Figure 17(a)). NL expression did not vary as a function of either infliximab concentration or the E:T ratio for either cells in culture or RTU cells (data not shown). Both the EC_50_ and dynamic range of the ADCC assay using the iLite effector cell line were superior to those obtained using a NFAT responsive reporter gene effector cell line when used in conjunction with the mTNF*α* HEK293 target cells (Figure 17(b)).

#### 3.2.11. Quantification of the ADCC Activity of TNF*α* Antagonists in the Serum of Patients with Autoimmune Disease

**Figure 18:**
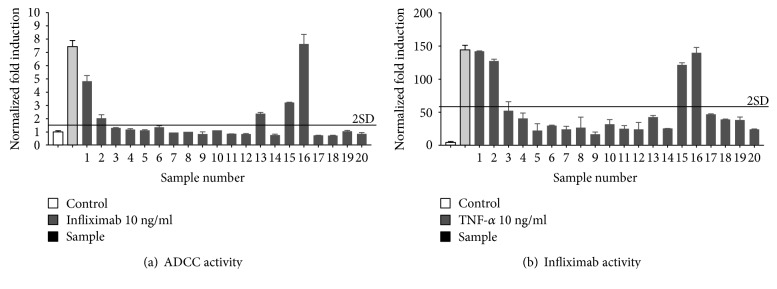
Quantification of the ADCC activity of infliximab in the sera of patients with Crohn's disease. iLite effector cells (1.2 × 10^5^ cells/well) were mixed with mTNF*α* target cells at an E:T ratio of 3 : 1 for 6 hours alone or in the presence of 10 ng/ml of infliximab or in the presence of an individual serum sample at a final dilution of 1/20 prior to the addition of Nano-Glo Dual-luciferase reagent (Promega, Madison, WI) and the sequential determination of FL and NL activity. Results are expressed as normalized fold ADCC activity relative to the control sample without infliximab (a). The same serum samples were analyzed for residual infliximab using an ELISA (Matriks Bioteck, Germany) or anti-TNF*α* activity in the presence of 10 ng/ml of TNF*α* using a reporter gene assay as described previously [23]. Results as expressed as normalized fold infliximab activity (b).

The level of nonspecific activation observed in the presence of a given concentration of human serum and the WT target cells that do not express membrane-bound TNF*α* (mTNF*α*) was determined and subtracted from that observed in the presence of the homologous cells expressing the specific drug target in the presence of the same concentration of human serum from patients with Crohn's disease treated with infliximab (Figure 18(a)). Samples 1, 15, and 16 that were found to exhibit ADCC activity were also found to contain infliximab using either an ELISA (Figure 18(b)) or anti-TNF*α* activity using a bioassay (not shown).

## 4. Discussion

Antibody-activated immune effector functions, in particular ADCC activity, are considered to play a determining role in the action of several therapeutic antibodies including rituximab, trastuzumab, and cetuximab [2]. Binding of an antibody to a specific antigen on target cells and the interaction of the Fc moiety of the antibody with the Fc*γ*RIIIA receptor on effector cells, including NK cells and other myeloid cells, leads to aggregation of Fc receptors on the immune effector cells and phosphorylation of immuno-receptor tyrosine-based activation motifs (ITAMs) on the intracytoplasmic domain of Fc receptors that share the conserved consensus sequence YXXLX_6-8_YXXL/I resulting in the release of cytokines, including IFN*γ*, and cytotoxic granules comprising perforin and granzyme, leading to cell lysis [15]. Phosphorylation of ITAM motifs by Src tyrosine kinases leads to activation of the Syk tyrosine kinase and calcium signaling [8–11, 16]. Activation of the calcium pathway by ITAM-coupled receptors results in the activation of the phosphatase calcineurin and dephosphorylation and activation of the transcription factor NFAT as well as phosphorylation and activation of CREB by a Ca2^+^/calmodulin-dependent protein kinase [9, 17]. The NFAT family of transcription factors bind DNA weakly and associates with other DNA-binding proteins including the transcription factors NF*κ*B and AP1 [8, 18, 19]. ITAM-coupled receptors have also been reported to interact with other signaling pathways including the Jak-STAT pathway [9]. In keeping with these results, we have shown that the NFAT, CREB, NF*κ*B, AP1, and the Jak-STAT pathways are all activated when Jurkat effector cells, cotransfected with a reporter gene construct specific for an individual transcription factor and CD16a, interact with CD20 positive Raji target cells in the presence of rituximab. The results of these experiments were used to design a chimeric promoter, carrying binding sites for NFAT, CREB, NF*κ*B, AP1, and STAT4/5, regulating transcription of the FL reporter gene that reflects more closely the Fc*γ*RIIIA signal transduction pathway, than a reporter gene construct regulated solely by a NFAT responsive promoter. Jurkat effector cells stably cotransfected with this reporter gene construct and CD16a exhibited increased sensitivity, specificity, and a larger dynamic range in an ADCC assay compared to Jurkat cells stably transfected with a 5-fold tandem repeat of the NFAT binding site alone regulating expression of the reporter gene construct [5, 6]. iLite effector cells expressing the V-158 variant of Fc*γ*RIIIA exhibited a higher ADCC activity than iLite effector cells expressing the F-158 allotype when tested with different therapeutic antibodies and target cells. These results are in keeping with the observation that the substitution of a phenylalanine in the F-variant for the valine at position 158 within the ligand binding domain of Fc*γ*RIIIA results in a lower binding affinity for IgG1, IgG3, and IgG4 and the observation that patients with non-Hodgkin's lymphoma homozygous for the F-158 allotype exhibit a lower response to treatment with the anti-CD20 monoclonal antibody rituximab than patients homozygous for V-158 allotype [20, 21]. Similarly, patients with metastatic colon cancer carrying the F-158 allotype were found to respond less favorably to treatment with the anti-epidermal growth factor receptor (EGFR) antibody cetuximab than patients homozygous for the V-158 allotype [22].

The choice of Jurkat cells, a human T-cell line, rather than a human NK cell line such as NK92 as the host cell line was dictated by the difficulty to transfect NK92 cells using conventional means and the biocontainment restrictions imposed by the alternative, lentiviral transduction.

The effector cell lines described herein that express firefly luciferase activity in response to ligation of an antibody to either the V-variant or F-variant of CD16a also express the Nano luciferase reporter gene under the control of a constitutive promoter. The activity of both firefly and Nano luciferase can be quantified sequentially in the same well of a microtiter plate allowing ADCC-activated FL expression to be normalized with respect to the constitutive expression of Nano luciferase. We have also shown previously that normalization of drug-induced reporter gene activity relative to the expression of an internal standardization gene, under the control of a constitutive promoter, renders assay results independent of cell number and provides a means for correcting for serum matrix effects and allows the activity of a biopharmaceutical to be quantified even in sera with a relative high level of cytotoxicity [23].

A series of novel target cells has been developed that expresses a constant high level of the specific antigen recognized by rituximab, trastuzumab, cetuximab, adalimumab, or infliximab under the control of a strong constitutive promoter as well as the homologous control target cells in which the gene encoding the specific antigen recognized by the therapeutic antibody has been inactivated by CRISPR/Cas 9 genome editing [12]. These engineered target cells and the homologous control cells allow differences in ADCC activity to be determined with precision. The expression of a constant high level of a specific antigen on the surface of the engineered target cells was associated with increased ADCC activity in the presence of the iLite effector cells and a specific therapeutic antibody, including rituximab, trastuzumab, and cetuximab, relative to that observed with the WT target cells that expressed a low endogenous level of the specific antigen on the cell surface. The availability of target cells specific for several of the most widely used therapeutic antibodies known to exhibit ADCC activity provides a precise means of comparing the ADCC activity of biosimilars with that of the innovator product as well as the comparison of the ADCC activity of variants of novel therapeutic antibodies that target the same antigen. The availability of target cells in which the specific drug target has been invalidated by genome editing provides the ideal control target cell for determining the specificity of an ADCC assay. In addition, the availability of target negative homologous control cells provides a very useful means of determining the level of nonspecific activation observed with effector cells and specific target cells in the presence of certain samples of human serum from normal donors or patients with autoimmune disease. The level of activation observed in the presence of the iLite effector cells and the target negative homologous control cells can be determined and then subtracted from that observed in the presence of the iLite effector cells and the specific antigen positive target cells as described in Materials and Methods. The same technique can also be used to distinguish between nonspecific activation and specific ADCC activity in samples of serum from patients with neoplastic or autoimmune disease treated with a therapeutic antibody. Thus, the use of this technique has revealed a close relationship between ADCC activity and infliximab levels, determined using a specific ELISA and infliximab activity determined using a TNF*α* responsive reporter gene assay [23] in archival serum samples from patients with Crohn's disease.

## 5. Conclusions

The development of novel effector cell lines that reflect more closely signal transduction from Fc*γ*RIIIa and novel target cells engineered to overexpress a constant high level of the specific antigen recognized by a therapeutic antibody has permitted the development of ADCC assays with improved sensitivity, specificity, and dynamic range relative to traditional ADCC assays using primary cells or effector cell lines that express a NFAT-regulated reporter gene and wild-type target cells. The availability of novel effector cells and target cells in a frozen thaw-and-use format, either separately or frozen together at the optimal E:T ratio, provides a convenient and cost-effective means of quantifying the ADCC activity of therapeutic antibodies. In addition, frozen RTU effector and target cells exhibit a low degree of vial-to-vial and lot-to-lot variation in their performance characteristics reflected by CVs of 10% or less for the principal parameters of a 4PL plot for an ADCC assay, in either a 96-well or 384-well plate format, and are ideally suited for a potency assay where reproducibility and precision are of paramount importance. Homologous control cells in which the specific target gene has been invalidated by genome editing provide an ideal control and a means of correcting for nonspecific effects observed with certain samples of human serum. Thus, novel effector cells and target cells expressing noncleavable membrane-bound TNF*α* have been used to quantify ADCC activity in serum from patients with Crohn's disease treated with infliximab and to relate ADCC activity to drug levels.
